# Multiple spatially related pharmacophores define small molecule inhibitors of OLIG2 in glioblastoma

**DOI:** 10.18632/oncotarget.5633

**Published:** 2015-10-30

**Authors:** Igor F. Tsigelny, Rajesh Mukthavaram, Valentina L. Kouznetsova, Ying Chao, Ivan Babic, Elmar Nurmemmedov, Sandra Pastorino, Pengfei Jiang, David Calligaris, Nathalie Agar, Miriam Scadeng, Sandeep C. Pingle, Wolfgang Wrasidlo, Milan T. Makale, Santosh Kesari

**Affiliations:** ^1^ Department of Neurosciences, University of California San Diego, La Jolla, CA, USA; ^2^ San Diego Supercomputer Center, University of California San Diego, La Jolla, CA, USA; ^3^ Translational Neuro-oncology Laboratories, Moores Cancer Center, University of California San Diego, La Jolla, CA, USA; ^4^ The Scripps Research Institute, La Jolla, CA, USA; ^5^ Harvard Medical School, Department of Neurosurgery, Brigham and Women's Hospital, Boston, MA, USA; ^6^ FMRI Research Center, Department of Radiology, University of California San Diego, La Jolla, CA, USA; ^7^ Current Address: John Wayne Cancer Institute at Providence Saint John's Health Center, Santa Monica, CA, USA

**Keywords:** in silico rational drug design, pharmacophore, inhibitor scaffold, transcription factors, OLIG2

## Abstract

Transcription factors (TFs) are a major class of protein signaling molecules that play key cellular roles in cancers such as the highly lethal brain cancer—glioblastoma (GBM). However, the development of specific TF inhibitors has proved difficult owing to expansive protein-protein interfaces and the absence of hydrophobic pockets. We uniquely defined the dimerization surface as an expansive parental pharmacophore comprised of several regional daughter pharmacophores. We targeted the OLIG2 TF which is essential for GBM survival and growth, we hypothesized that small molecules able to fit each subpharmacophore would inhibit OLIG2 activation. The most active compound was OLIG2 selective, it entered the brain, and it exhibited potent anti-GBM activity in cell-based assays and in pre-clinical mouse orthotopic models. These data suggest that (1) our multiple pharmacophore approach warrants further investigation, and (2) our most potent compounds merit detailed pharmacodynamic, biophysical, and mechanistic characterization for potential preclinical development as GBM therapeutics.

## INTRODUCTION

Transcription factors (TFs) comprise a large class of proteins that bind specific DNA regions and regulate gene expression to control key processes such as differentiation, the cell cycle, survival, and apoptosis [1, 2]. TF dysregulation is implicated in many disorders including cancer where TFs can act either as tumor suppressors or oncogenes [3, 4]. Moreover we have previously reported that specific TFs can affect genes related to brain development [5] and potentially impact post-natal brain developmental disorders. Hence TFs may represent an important therapeutic target [6–9]. However, the development of specific inhibitors has proved difficult owing to large TF protein-protein dimerization interfaces, the absence of hydrophobic pockets, and different TF conformations between free and dimerized states [10].

Direct inhibition of TFs would ideally involve a small molecule that sufficiently interferes with TF-DNA interaction or with TF-activating dimerization [11]. Importantly, these two avenues have led to some success. For example, a small molecule can inhibit binding of the TF C/EBPα to DNA [12, 13]. Similarly, compounds have been shown to inhibit c-Myc/Max heterodimerization and hypoxia-inducible factor 1α (HIF1α) and coactivator p300 (or CREB binding protein) [14–16]. The limited results so far attained suggest that TFs can potentially be directly inhibited.

Considering the biological importance of TFs and their role in the growth and proliferation of cancer stem-like cells [17–19], novel strategies to design small-molecule TF inhibitors are needed. *In silico* modeling is increasingly being used in rational drug design, but previous *in silico* based attempts to design TF inhibitors have largely failed. Our analyses indicate that this failure resulted from the erroneous assumption that one key discrete site is present in the dimerization interface and that this relatively small locus—termed a binding “hotspot”— can be relied upon to guide the design of inhibitory scaffolds [11, 20, 21]. In contrast, our computational analyses suggested that in actuality the active TF dimerization surface includes a comparatively much larger engagement area we define as the parental pharmacophore, which is in turn comprised of several distinct daughter pharmacophores (subpharmacophores) with identifying features. We have previously successfully applied this multiple pharmacophore concept for defining ligand-based pharmacophores [22–23] and interface pharmacophores [24] to drug-candidate development.

We pursued our multiple pharmacophore concept for the OLIG2 TF dimerization interface. OLIG2 is a basic helix-loop-helix (bHLH) TF that is critical in tumorigenesis and regulates the survival and expansion of glioblastoma (GBM) [25–30]. Our objective was to define the OLIG2 dimerization pharmacophore complex and search existing chemical structure libraries for compounds predicted to engage all the daughter pharmacophores. Such an agent could in principle populate all the essential elements of the dimerization surface and thus inhibit or interfere with proper dimerization and TF activation. We validated this approach by demonstrating the OLIG2 pathway selectivity and potent *in vitro* anti-GBM activity of *in silico* identified compounds.

A key challenge with many transcription factors including OLIG2 is that high-resolution crystal structures are not available. However, OLIG2 is known to bind E47, one of the isoforms of E2A class TFs for which a crystal structure is resolved [31]. In addition, OLIG2 has close sequence identity to a number of other TFs that bind the E2A isoforms, E12 and E47. Based on this information, we analyzed possible intermolecular contacts between OLIG2 and E2A isomers, and focused on the NeuroD1 TF, which has very close sequence identity to OLIG2. Using the E47-NeuroD1 complex as a template [32], we modeled OLIG2 and the OLIG2-E47 heterodimer, allowing the novel definition of a combined pharmacophore hypothesis comprised of one parental and multiple daughter pharmacophores.

Here we demonstrate how our combined pharmacophore guided 3D-structure searches of the Open NCI Database (http://cactvs.nci.nih.gov/download/nci/) to identify compounds potentially able to engage the OLIG2 dimerization surface. Compounds predicted to engage with all three hypothesized OLIG2 daughter pharmacophores were screened *in vitro* against patient-derived GBM tumorspheres. We found several small molecules that potently suppressed the growth of GBM tumorspheres *in vitro*, and the most effective candidate, SKOG102, also markedly attenuated human tumors in two pre-clinical *in vivo* GBM models. SKOG102, which enters the brain after intravenous injection, selectively modulated downstream OLIG2 targets, and downregulated stem cell and oligodendrocyte lineage markers to the same degree as shRNA-mediated OLIG2 knockdown. These results underscore a potential to pharmacologically suppress the stem cell-like tumor compartment presumed to drive GBMs. The data presented herein provide a basis and impetus for subsequent detailed biophysical explorations of the nature and timescale of the engagement of SKOG102 with the OLIG2 transcription factor, in order to facilitate its optimization as a potential OLIG2 inhibitor for GBM and other CNS diseases.

## RESULTS

### Homology modeling to develop a template for OLIG2 dimerization

**Figure 1:**
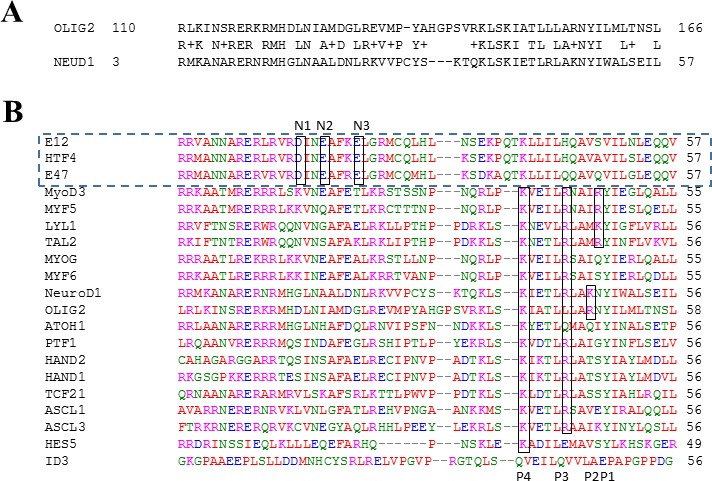
Sequence alignment of transcription factors relevant to OLIG2 **A**. OLIG2 and NeuroD1; **B**. Sequence alignment of transcription factors that we called “E2A set” (outlined by the dashed rectangle) including E12, E47, and HTF4 with negative residues N1-N3 in the interface region and the TFs complementary to them; P1-P4 are positive residues in the interface area.

**Figure 2:**
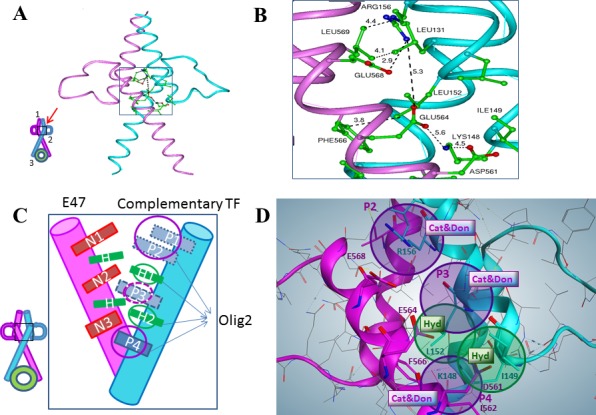
Homology modeling and definition of the OLIG2 pharmacophore **A**. General structure of OLIG2 (cyan)-E47 (magenta) heterodimer, inset depicts the topological scheme of the interface, 1-upper interface, 2-middle interface (red arrow indicates the region of pharmacophore design), and 3-interface with DNA. **B**. Close-up of the middle interface interactions. One can see locations of key residues involved in the specific interaction between OLIG2 and E47 and distances between them. The interaction zone includes the E47 negative residues Asp561, Glu564, and Glu568 and the OLIG2 positive residues Lys148 and Arg156. **C**. Scheme of the OLIG2-E47 interface created by TF features. Details in the area depicted by the rectangle are presented in the zoomed-in view. Complementary combinations to E47: set 1 (S1, OLIG2 or NeuroD1)-P2, H1, H2, P4; set 2 (S2)-P1, P3, H1, H2, P4; set 3 (S3)-P2, P3, H1, H2, P4. This organization leads to the definition of the main features of four pharmacophores including the parental and 3 daughters: Pharmacophore 0 (five features, parental): P1 or P2; H1; P3; H2; P4; Pharmacophore 1 (daughter, four features): P1 orP2; H1; H2; P4 (OLIG2 and similar); Pharmacophore 2 (daughter, four features): P1 or P2; H1; P3; H2; Pharmacophore 3 (daughter, four features): H1; P3; H2; P4. **D**. Pharmacophore hypothesis mimicking OLIG2 complementary interaction. Purple spheres P2 and P4 are Cationic and Donor (Cat&Don) centers based on Arg156-Glu568 and Lys148-Asp561 positive-negative interactions; purple sphere P3 (Cat&Don center) added to bind E47's residue Glu564. Green spheres are Hydrophobic (Hyd) centers based on Leu152-Phe566 and Ile149-Ile562 hydrophobic interactions.

In order to model 3D structure and the OLIG2-E47 dimerization interface, homology modeling of OLIG2 was conducted. We also analyzed possible structures of the heterodimers of E47 with other TFs similar to OLIG2, included in the alignment shown in Figure 1B (set of TFs below the dashed rectangle). The general scheme of the interface between the group containing E2A isomers and HTF4 TFs (the E2A set) is outlined by the dashed rectangle in Figure 1B). Based on strong homology between OLIG2 and NeuroD1, we modeled the 3D structure of the OLIG2-E47 heterodimer (Homology program, InsightII package, Accelrys, San Diego, CA) using the crystallographic structure of the NeuroD1-E47 heterodimer as a template (PDB ID: 2ql2; Figure 1A; [32]). Our modeled OLIG2-E47 dimer structure is depicted in Figure 2A, with the inset illustrating the general topology of the heterodimer. This structure contains unique sequence that can be a basis for assigning pharmacophore features, and there are three contact areas: 1 and 2 between member monomers and 3 with DNA. The E47 negative amino-acid residues—aspartic acid (Asp561), glutamic acid (Glu564 and Glu568) interact in close proximity with the OLIG2 positive amino-acid residues lysine (Lys148) and arginine (Arg156) (Figure 2B).

The OLIG2-E47 interface has a unique profile and is amenable to the design of selective agents, although there are three conserved negative residues—one aspartic acid (N3) and two glutamic acid (N1 and N2) residues—in the E2A group, two positive residues—lysine (P4) and arginine (P3)—conserved among most of the other complementary TFs, and a third positive site located farther in the sequence and conserved in specific sets of TFs (including P1 and P2) (Figure 2C). All these residues are marked and outlined by the rectangles on the alignment depicted in Figure 1B. Based on these data, three complementary combinations to E47 could be considered for pharmacophore development and the scheme of the E47 interface with a complementary TF, including OLIG2, is shown in Figure 2C. There is evident complementarity of the negative residues on E47 (N1—N3) to the positive residues (P1—P4) on the partner TF. Based on this reasoning three combinations complementary to E47 should be considered for pharmacophore development: set 1 (S1) OLIG2 or neuroD1 - P2, H1, H2, P4; set 2 (S2) P1, P3, H1, H2, P4; and set 3 (S3) - P2, P3, H1, H2, P4. Importantly, two possible positions of less conserved positive residues for S1 and S2 are not far from each other in the 3D structure (see Figure 2B and 2D). Their side chains can have conformations that will bring them into the attracting energy distance as exemplified by the negative residue Glu568 (N1) belonging to E47 interacting with the residue Arg156 (P2) (see Figure 2B and 2C). Figure 2D shows a pharmacophore model developed using the aforementioned considerations.

### OLIG2 pharmacophore modeling

#### Parental pharmacophore

**Figure 3:**
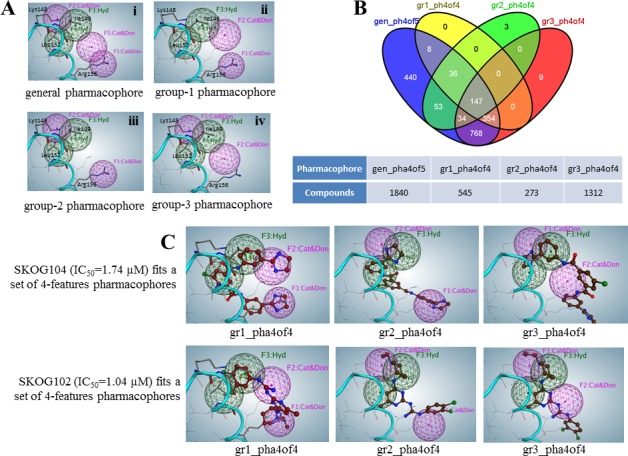
Parental and daughter pharmacophore definitions that guided conformational database searches **A**. Five-feature parental (*i*) and four-feature daughter (*ii*, *iii*, and *iv*) pharmacophores. Ribbon diagram and residues presented by lines belong to the superimposed OLIG2 protein. **B**. Venn diagram for four sets of compounds identified from a four pharmacophore-hypotheses based search in conformational database derived from the Open NCI compounds *in silico* library. For example, using the gr1 daughter pharmacophore the program selected 545 compounds from which 147 were also selected using gr2, gr3, daughter and the parental (gen) pharmacophore. **C**. Two compound examples, SKOG104 and SKOG102, are theoretically able to engage all three subpharmacophores within the dimerization region. The individual figure panels illustrate the predicted fit of SKOG102/4 to the three daughter pharmacophores.

The previous section outlines the basis for the development of pharmacophores for TFs complementary to E47 and this approach was used to define pharmacophores for OLIG2. Here the combination of interactions for group S2 includes five interactions: three negative-positive and two hydrophobic, which could be covered with S1 and S3. Hence the pharmacophore for S2 can be considered as parental and the rest as daughter pharmacophores. TFs similar to OLIG2 (see Figure 1) can be separated into three sets depending on their sequences (see previous section); so a daughter pharmacophore for each set can be developed. The purple spheres shown in Figure 3A and 3C represent cationic/donor features, while the green spheres denote hydrophobic features of the pharmacophore. The parental five-feature pharmacophore hypothesis (Figure 3A, panel *i*) included all cationic/donor and hydrophobic features.

#### Subpharmacophores (daughter pharmacophores)

Binding E47 with TFs corresponding to the sets S1 and S3 is directed only by two positive-negative contacts of all three such possible contacts in each case. So we created a set of four-feature daughter pharmacophore hypotheses containing the various combinations of pairs of cationic/donor features with the same two hydrophobic regions (Figure 3A). Using the set of daughter pharmacophores derived from a parental pharmacophore and having a smaller number of features allows decreasing the size of possible compounds obviates the need for large molecules that can have difficulty crossing the blood-brain barrier.

### Pharmacophore-guided structure similarity searches of conformational databases

The parental and daughter pharmacophores were used to virtually search our conformational databases derived from the Open NCI Chemical Structure Database (release 3, accessed April 2012) of 260,071 compounds. We excluded compounds with molecular weight > 600 Da and of these the search yielded 1840 compounds (1.3% hit rate) predicted to fit 4 of 5 features belonging to the parental pharmacophore. Subsequent searches based on the 3 daughter pharmacophores, utilizing 4 features, yielded sets of 545, 273, and 1312 compounds, termed group-1 (gr1), group-2 (gr2), and group-3 (gr3), respectively. The four-set Venn diagram in Figure 3B reveals that there are 147 compounds theoretically predicted to fit the 4-of-5 features of the parental and all features of daughter pharmacophores. It is noteworthy that the compounds selected from the Open NCI Database set, which showed the most potent activity in subsequent GBM cell-based screens, were mainly those that were predicted to fit all hypothesized pharmacophores. Figure 3C shows the possible configurations of two selected compounds that fit all four-feature subpharmacophores. Both of these compounds exhibited comparatively potent anti-GBM activity.

### Definition of compound structure classes

The next step was to elucidate the common structural and chemical properties and features of the selected compound subsets for *in vitro* validation of pharmacophore-based predictions and for the subsequent development as GBM therapeutics. The compounds that fit all our 4-feature pharmacophores were clustered into five discrete structural class clusters (A—E), such that each of the cluster classes consisted of at least five compounds, and 76 compounds deemed tractable were included in these clusters (Dataset S1). Descriptions of structural classes were given below:

Cluster A—Includes 23 compounds that have the quininoline moiety in common. These compounds are frequently asymmetric, having an aliphatic or alicyclic tail terminating in a substituted amino group.
Cluster B—Contains 26 compounds all of which are either aromatic amides or ureas and most frequently have terminal dihydroimidazole ring structures.
Cluster C—All 5 compounds from this cluster can be classified as polyphenolic and are terminated by trisubstituted amino groups.
Cluster D—All 16 compounds in this cluster have either terminal substituted guanidine groups or disubstituted guanidine groups in the center of the molecules.
Cluster E—All 6 compounds in this cluster have the acridine moiety as a central scaffold and are substituted with hydrogen bond donating amino groups.

### Biochemical and cell-based validation of compounds identified by pharmacophore modeling

#### In vitro anti-GBM activity of candidate compounds

**Figure 4:**
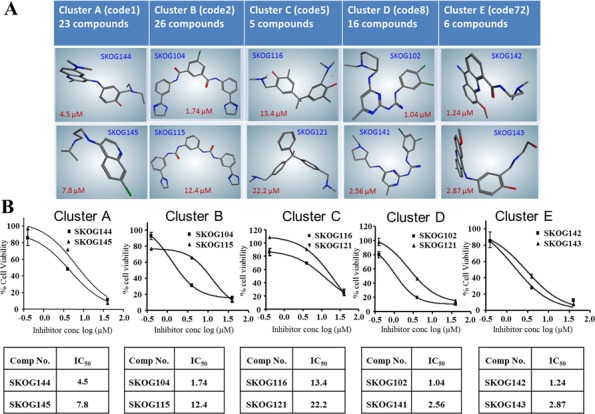
Structural classes of potential OLIG2 inhibitors and *in vitro* anti-GBM potency Figure depicts structural classes and representative scaffolds **A**. while **B**. shows the associated IC_50_ curves for *Ink4a/arf* EGFR-VIII cells treated with the two most potent compounds from each structural class, or cluster. In addition to the 75 compounds belonging to the 5 classes, another 20 disparate structures were also tested, and these showed little activity. Error bars represent mean ± standard deviation.

**Figure 5:**
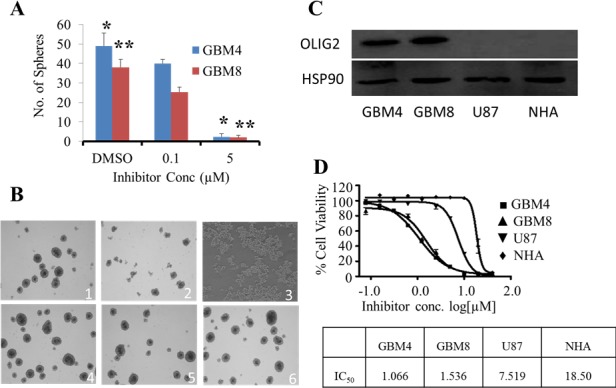
Compounds selected by subpharmacophore modeling-driven database searches were selective for OLIG2 **A**. One of the most potent OLIG2 inhibitors identified by our modeling methodology clearly inhibits human GBM4 and GBM8 cells grown as neurospheres **B**. in a dose-dependent fashion. DMSO was the vehicle control. *, ** indicate *p*-values between control and 5 μM SKOG102 treated cells, * for GBM4 cells *p* = 0.0003 and for GBM8 cells *p* = 0.001. **C**. Western blot data for OLIG2 expression in patient-derived GBM lines (GBM4 and GBM8), in an immortalized, serum-grown GBM cell line (U87), and in normal human astrocytes (NHA) freshly acquired from patient material. The blot image was prepared by cropping out middle lanes, the gel and blotting conditions were identical for the lanes shown in the figure. **D**. The GBM4/8 GBM lines express OLIG2 and exhibit an IC_50_ between 1 and 2 μM. U87 cells, which express much less OLIG2 than GBM4/8 cells, were also suppressed, but a roughly 7-fold higher dose was needed. NHA, which express no detectable OLIG2, had an IC_50_ of about 20 fold greater than GBM4 cells. All samples run in duplicate. Error bars represent mean ± standard deviation.

Twenty-two compounds of the 95 tested exhibited considerable potency against GBM cells *in vitro*, and were screened initially using tumorigenic *Ink4a/arf* EGFR-VIII mouse glioma stem cells because these cells express high levels of OLIG2 [33]. The results reveal that the predictive performance of the multiple pharmacophore approach was 23.16%. Figure 4 shows the representative clusters of structurally related compounds and their associated IC_50_ data, and the figure reveals that the compound identified as SKOG102 exhibited the greatest potency. SKOG102 inhibited sphere formation of two patient-derived GBM lines that are routinely cultured as tumorspheres (Figure 5A and 5B). These GBM tumorspheres are propagated from GBM stem-like cells isolated from primary patient material and are highly representative of the phenotypic heterogeneity, compartmentalization, and behavior of the original tumors [26–28].

#### Lead inhibitor compound selectivity for OLIG2

The lead compound SKOG102 exerted potent cytotoxicity only in those cell lines expressing OLIG2, suggesting that it acted primarily via the OLIG2 pathway. Western blot and RT-PCR analysis showed that GBM4/8 cells expressed significantly more OLIG2 than U87 cells while normal human astrocytes (NHA) expressed no OLIG2 (Figure 5C and Figure S 1). The plots in Figure 5D clearly show that the IC_50_ of SKOG102 in GBM4/8 cells was much lower than in U87 cells. Additionally, NHA required a very high dose of the inhibitor for toxicity to appear.

**Figure 6:**
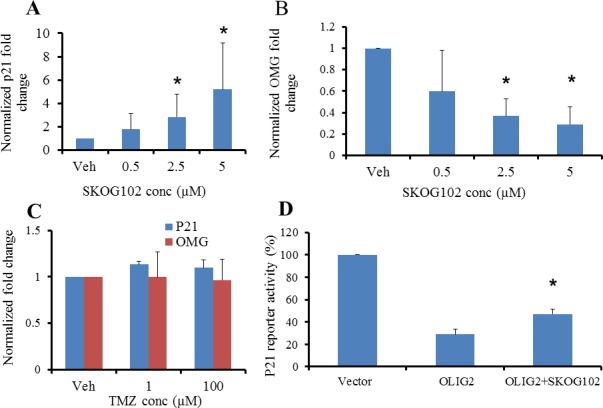
OLIG2 inhibitor affects expression of direct genetic targets of OLIG2 The expression of p21 and OMG were determined by RT-PCR. **A**. Escalating OLIG2 inhibitor doses led to increased levels of p21. **B**. Increasing concentrations of OLIG2 inhibitor suppressed the expression of OMG. **C**. GBM4 cells were treated with vehicle or TMZ (1 or 100 μM) for 12 h followed by RT-PCR analyses to determine p21 and OMG expression. * *p* <0.01 between vehicle control and SKOG102 treated groups. **D**. SKOG102 derepresses OLIG2 mediated repression of luciferase activity in p21 reporter assay. *p* = 0.0089. All these results suggest that the OLIG2 inhibitor compound specifically targets OLIG2.

As a further indication of compound selectivity we examined whether the expression of direct genetic targets of OLIG2 varied with inhibitor dose. Escalating doses of SKOG102 caused p21 RNA to increase in a dose-dependent manner (Figure 6A). In addition we tested one compound from each cluster: SKOG145 (cluster A), SKOG109 (cluster B), SKOG108 (cluster C), and SKOG142 (cluster E)—on GBM4 cells to analyze p21 gene expression, where the OLIG2 inhibitor was added in escalating doses. As with SKOG102, the levels of p21 increased in a dose dependent manner (Figure S 2). Conversely to p21, OMG is upregulated by OLIG2 and we found that SKOG102 caused OMG RNA levels in GBM4 to decline, again in a dose dependent manner (Figure 6B). We asked whether the current front line GBM therapeutic Temolozomide (TMZ), which is a general DNA alkylating agent, would have any effect on p21 and OMG expression. Both 1 μM and 100 μM TMZ had a negligible effect on p21 and OMG expression in GBM4 cells (Figure 6C). The clear implication is that SKOG102 acts via a different pathway than TMZ, and this is important because as is well known in the field, TMZ is the front line GBM therapeutic and hence new therapeutics should avoid redundancy. Using a p21 promoter luciferase reporter we observed SKOG102 increased expression of the reporter expression, indicating that SKOG102 directly affects OLIG2 function (Figure 6D). SKOG102 derepressed OLIG2 mediated repression of luciferase activity in a p21 reporter assay strongly suggesting that the OLIG2 inhibitor compound specifically targets OLIG2 pathway (Figure 6D).

To further profile OLIG2 engagement by SKOG102 we sought to determine whether SKOG102 is directly engaging OLIG2 and interferes with the ability of OLIG2 to bind DNA. For a determination of SKOG102 - OLIG2 engagement we employed a thermal shift assay. The protein purity was confirmed by a third party (Blue Sky Inc.) (Figure S 3) and the stability profile for His-tag OLIG2 protein was calculated, (Figure S 4), and we then performed the thermal shift assay using His-tag purified OLIG2 protein and SKOG102. Shifts in melting temperature of the protein without and with the SKOG102 are summarized in Table1 and melting curves are shown (Figure S 5). OLIG2 displays a significant shift in Tm in response to increasing concentrations of the inhibitor SKOG102. At 12.5 μM, Tm shifted by 4.1°C with a single minimum. At 25.0 μM, a second minimum emerged indicating a new population of inhibitor-stabilized protein, with an average ΔTm of 7.5°C. At 50 μM, all of the protein is saturated with the inhibitor displaying ΔTm of 12.0°C. Transition of the protein melting profile from low to high Tm suggests stabilization of the protein structure by the inhibitor. The calculated affinity of SKOG102 interaction with OLIG2 at the highest saturating concentration is 56 nM. Importantly, this study was not intended to elucidate the role of E47:OLIG2 binding, but rather the aim was to disrupt the OLIG2 pathway by interfering with the behavior of OLIG2.

**Figure 7:**
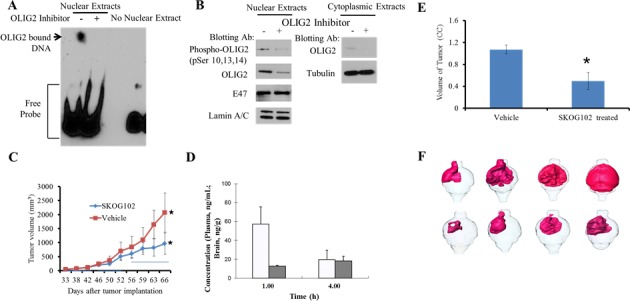
SKOG102 suppresses OLIG2-DNA binding, it enters the brain, and it inhibits GBM *in vivo* growth GBM8 patient-derived cells were treated with vehicle or 5 μM Olig2 inhibitor for 20 hr. **A**. Nuclear fractions were isolated and used for an electrophoretic mobility shift assay (EMSA). The full length blot is shown in Figure S 10 in Supplementary Materials. **B**. Nuclear fractions were probed for phosphorylation of OLIG2 and total OLIG2. Cytoplasmic extracts were also examined for re-localization of OLIG2 from the nucleus to the cytoplasm. OLIG2 does not re-localize to the cytoplasm after inhibitor treatment. Each panel was run under the same conditions and the full length blots are shown in Figure S 11 in Supplementary Materials. **C**. Flank tumor study. GBM4 cells were implanted subcutaneously into NSG mice, dosing was started when tumors were palpable. Dosing schedule indicated by horizontal bars on graph was first 2 weeks (from day 33 to 46) 10 mg/kg, third week (from day 47 to 54) 15 mg/kg, fourth week (from 55 to 66) first 3 days 20 mg/kg, 2 days break, then alternate days from day 60 to 66. GBM4 tumor growth was inhibited after treatment with SKOG102 when compared vehicle treated group. Error bar represent mean standard deviation, * indicates *p* = 0.02 between vehicle control and SKOG102 treated group. **D**. Brain concentration of SKOG102. Mice were injected with 5 mg/kg intraperitoneally and the graph indicates the brain concentration at 1 and 4 hours. **E**. Intracranial tumor study. GBM4 cells were pretreated with SKOG102 or vehicle control for 14 h, only viable cells were injected intracranially into NSG mice (*n* = 8). Tumor volumes were measured using MRI after 4 weeks. Bar graph indicates tumor volumes, error bars represent mean ± standard deviation, * indicates *p* < 0.05 between vehicle control and SKOG102. **F**. Renderings of mouse brain tumors imaged with contrast enhanced MRI, where cells were pretreated with DMSO vehicle (upper panels) and SKOG102 (lower panels), and viable cells were injected intracranially into NSG mice. Area of the enhanced relative intensity was color coded to produce a 3D model of the extent and distribution of the tumor cells in the brain tissue.

We determined whether SKOG102 impacts OLIG2 DNA binding ability. This was pursued via an electrophoretic mobility shift assay (EMSA). Patient-derived GBM8 cells were treated with SKOG102 or vehicle alone. Nuclear extracts were isolated and examined for binding to DNA harboring an OLIG2 consensus motif. SKOG102 treatment abrogated protein-DNA interaction (Figure 7A). Surprisingly, we observed a decrease in serine phosphorylated OLIG2 and total OLIG2 protein levels in SKOG102 treated patient-derived cells (Figure 7B). E47 protein levels were not decreased in the nuclear fraction of SKOG102 treated cells demonstrating specificity of SKOG102 to OLIG2 (Figure 7B).

#### Activity against GBM cancer stem cells

The goal here was to determine whether OLIG2 inhibition could suppress the cancer stem cells that drive GMB initiation and expansion. Patient derived cancer stem cell tumorspheres grown *in vitro* had OLIG2 either knocked down with shRNA (Figure S 6) or pharmacologically inhibited with SKOG102. These two treatments similarly affected the expression of stem cell markers nestin and CD133, and multiple oligodendrocyte lineage markers 2′,3′-cyclic-nucleotide 3′-phosphodiesterase (CNPase), myelin-basic protein (MBP), oligodendrocyte myelin glycoprotein (OMG), and proteolipid protein 1 (plp1) (Figures S 7 and S 8). These results suggest a potential for OLIG2 inhibitors such as SKOG102 to differentiate the GBM cancer stem-like cellular compartment to a state in which it is less capable of promoting tumor survival and expansion.

#### Suppression of GBM in vivo

In this part of the study we sought to determine whether pharmacologic OLIG2 inhibition could potentially suppress GBM expansion *in vivo*. Initially we performed xenograft flank tumor experiments in which the mice were inoculated in each flank with GBM4 cells. Dosing with the most potent compound from structural class I—SKOG102—at 10, 15, and 20 mg/kg was performed biweekly for three weeks and the 20 mg dose significantly attenuated tumor growth (Figure 7C). The key observation is that the highest dose, 20 mg, has a marked effect on tumor size.

Next we extended the *in vivo* studies to a GBM patient derived cancer stem cell (GBM4 line) based orthotopic model that accurately emulates the invasive natural history of the human disease. First we sought to confirm that SKOG102 could enter the brain so a test dose of 5 mg/kg was injected intraperitoneally into intact balb/c mice, so at 1 and 4 hours the plasma and brain concentration of SKOG102 was determined using LC-Mass spec. The compound did accumulate in the tumor (Figure S 9) even over a relatively brief time period and the plasma and brain concentrations were almost identical at 4 hours after injection (Figure 7D), while extrapolation to higher doses and regimens with multiple dosing (15 injections of 100 mg/kg) suggests that a potent therapeutic dose can be attained, ≈ 12.6 μM in which proliferation was suppressed by almost 90% (Figure 4B; cluster D). After the dosing study equal numbers of GBM4 cells pretreated with SKOG102 or vehicle control (DMSO) were inoculated into the right frontal cortex of NSG SCID mice. After 4 weeks MRI volumetry showed that SKOG102 pretreated cells formed tumors that were on average 50% smaller than vehicle control treated cells (Figure 7E and 7F). It should be note at this juncture that knockdowns of OLIG2 are lethal, particularly in patient derived lines, which makes rescue study not possible. We employed OLIG2 siRNA and SKOG102 neither of which are technically amenable to rescue studies.

## DISCUSSION

The development of protein interface pharmacophores has thus far proved virtually intractable and this reality has limited the development of TF inhibitors [34]. The challenge involved with TFs is particularly evident when compared with most typical drug-design scenarios, which usually involve designing so-called “pocket” inhibitors that fit within the well-defined boundaries of a protein pocket within a target protein. Such a pocket constrains the possible positions of the prototype compound, critical reference points relating to shape, and all necessary pharmacophore features corresponding to the residues of the pocket. In contrast, large protein-protein interfaces such as those presented by TFs contain no such boundaries and no shape limitations. The involved protein surfaces are wide open in terms of a solution and have a comparatively “flat” shape that is not amenable to selectivity of peptide or small molecule inhibitors [10].

In view of the foregoing we introduced the concept of a “combined pharmacophore hypotheses,” which is embodied by a computationally derived set comprised of a “parental pharmacophore” and multiple “daughter pharmacophores.” A set of combined pharmacophores was used to search *in silico* libraries including the NCI open database, for single structures able to engage all proposed pharmacophores and thus reasonably selectively populate the OLIG2 dimerization surface at enough points to effectively disrupt OLIG2 function. This methodology could potentially be generalized to various TFs and other protein-protein interface targets. Importantly in this context, our work elucidated the salient features not only of the OLIG2-E47 interface, but also of a number of bHLH heterodimers having OLIG2-E47 related sequences and structural identities. Notwithstanding, it is important to recognize that our modeling assumed that OLIG2 was in a dimerized state and bound to DNA. Therefore precisely at what point in the dimerization and DNA binding steps SKOG102 and other identified candidates engage OLIG2, and whether this is a permanent or transient interaction, is unknown and will likely complicate studies of OLIG2 - SKOG102 binding. Pertinent biophysical and X-ray crystallographic studies are planned but these will be of a comprehensive long-term nature, and as such were considered to be beyond the scope of the present exploratory report.

We validated the combined pharmacophore approach by an array of methods including, (1) quantifying the anti-tumor cell potency of *in silico* identified compounds with cultured human GBM cell lines, (2) demonstrating that cell lines expressing OLIG2 showed a relatively much greater susceptibility to the OLIG2 inhibitor, and (3) showing selective inhibition of OLIG2 by measuring OLIG2 target gene effects by quantitative RT-PCR, reporter assays, a thermal shift assay, and EMSA. Importantly, both the flank tumor study and the orthotopic study showed that SKOG102 treatment reduced tumor growth by more than 50% compared with controls. In the flank tumor studies we only used 20 mg/kg of SKOG102 and did not escalate the dose to higher levels. We were looking for activity, and considering that the current GBM front line agent TMZ is given on the order of 250 - 400 mg/kg, achieving a 50% reduction in tumor size with 20 mg/kg of SKOG102 is noteworthy. Moreover for the orthotopic model in which the tumorspheres received only one round of SKOG102 before implantation there was also a 50% reduction in tumor size which is impressive considering that the tumors were not exposed to SKOG102 after being implanted.

Our combined pharmacophore approach is potentially applicable to other TFs, a class of proteins that has generally proved very difficult to selectively inhibit with small molecule scaffold designs. The data presented herein warrants subsequent long-term, detailed biophysical and mechanistic determinations of precisely how the most potent compounds we identified cause OLIG2-selective dysfunction. In future studies it would be of interest to characterize GBM stem-like cells and their phenotypic response to OLIG2 inhibition. SKOG102 and other compounds that exhibited anti-GBM activity are chemically tractable and suitable for structural modification to (1) enhance OLIG2 selectivity which may be important as other targets in addition to OLIG2 may be variously engaged by SKOG102 in its current form, and (2) optimize pharmacokinetics and the toxicity profile of SKOG102, and assess its survival extending effect, in order to pursue the development of this scaffold as a potential GBM and CNS therapeutic.

## MATERIALS AND METHODS

### Homology and pharmacophore modeling

As the first step in our analyses, we selected TFs that bind to E2A isoforms using the APID program [35]. The sequences of the selected TFs were aligned with the program Clustal W 2.1 [36]. The next step involved preparing a homology model of OLIG2 and its heterodimer with E47, using the InsightII package (Accelrys, San Diego, Calif.). As a basis for this modeling, we used the known structure of the E47-NeuroD1 dimer (PDB ID 2ql2). We modeled only the specified region of interest in OLIG2 that was previously selected on the basis of alignment (Figure 1A). In this region the sequence homology of OLIG2 to NeuroD1 was found to be ~55% using the homology module of InsightII^®^ modeling software. The resultant modeled OLIG2-E47 structure then underwent 10,000 iterations according to molecular mechanics minimization using the Discover program (Accelrys, San Diego, Calif.).

Our parental pharmacophore hypothesis included two hydrophobic and three cationic/donor features. Based on this hypothesis, three different four-feature daughter hypotheses were designed, each of them containing the same two hydrophobes and different combinations of two of three cations/donors.

The Open NCI Database (http://cactvs.nci.nih.gov/download/nci/) release 3 (260,071), containing 3D structures of over 250,000 compounds was searched for compounds that would fit all pharmacophores to create the four-set Venn diagram, using the VENNY server [37]. Fingerprints for the compounds from the intersection zone common to all four sets were calculated and the compounds were clustered using the similarity method (MOE Fingerprints module). Then we applied nearest-neighbor Jarvis-Patrick clustering with similarity (*S*) and overlap (*O*) parameters: *SO* = 0.45 [38, 39], and the Tanimoto coefficient, as similarity metrics to create compound structural classes [40, 41].

### Cell culture and cell viability assays

#### Culture and assay system

*Ink4a/arf* EGFR-VIII mouse cells and U87 human GBM cells were cultured in DMEM medium with 10% FBS. GBM4 and GBM8 patient-derived tumor neurosphere lines were cultured in stem cell medium supplemented with FGF and EGF. Primary normal human astrocytes (NHA) were cultured in astrocyte medium (Life Technologies, Grand Island, N.Y.) with EGF. Cytotoxicity of the compounds was assessed by 3-(4,5-dimethylthiazol-2-yl)-2,5-diphenyltetrazolium bromide (MTT) reduction assay (for *Ink4a/arf* EGFR-VIII cells) as described earlier [42] or by Alamar Blue assay (for all other cell types).

For GBM4, GBM8, U87, and NHA cell lines, viability was quantified by Alamar Blue Assay. In this assay, cells were treated with inhibitor compounds as described and Alamar Blue added after 72 h. Emission values at 590 nm were measured after the addition of Alamar Blue. Dose-response curves for MTT assays and Alamar Blue Assays were plotted and IC_50_ values were calculated by using GraphPad Prism (GraphPad Prism Software, Inc., La Jolla, Calif.).

### Chemosensitivity of GBM neurospheres

GBM4 cells were plated in 96-well plates and cultured as neurospheres [43]. The active compound SKOG102[1-(3,4-dichlorophenyl)-3-(4-((1-ethyl-3-piperidyl)amino)-6-methyl-2-pyrimidinyl)guanidine] was added at varying concentrations 12 h after plating. Neurospheres were viewed and photographed under Nikon microscope 4× objective after 72 h of incubation.

### Quantitation of OLIG2 and OLIG2-target mRNA expression

Cancer stem-like cells were isolated from patient GBM tissue samples and cultured in NSA proliferation medium (Stemcell Technologies, Vancouver, Canada). After 72 hour incubation with control shRNA or OLIG2 shRNA, or after 12 h of incubation with DMSO control or OLIG2 inhibitor compound or temezolomide, cells were harvested and mRNA was extracted with the AllPrep DNA/RNA Mini Kit (Qiagen, Inc., Valencia, CA). This was followed by cDNA synthesis using the iScript cDNA Synthesis Kit (Bio-Rad, Inc., Hercules, CA). To investigate single-gene expression patterns, individual gene primers were identified in the PrimerBank database (http://pga.mgh.harvard.edu/primerbank/) and then were purchased from Allele Biotechnology and Pharmaceuticals Inc. (San Diego, CA). SYBR Green Real Time PCR master mixes were purchased from Roche Corporation (Roche New Jersey, Nutley, N.J. or Roche Madison, Madison, WI) RT-PCRQT-PCR was performed with primers specific for human OLIG2, p21, OMG, CD133, Nestin, MBP, CNPase, and PLP1 genes. Individual gene expression was normalized to the expression of β-Actin. OLIG2 expression in U87 cells, normal human astrocytes, GBM4, and GBM8 cells was quantified by immunoblottingwith OLIG2 antibody (kindly provided by Charles Stiles's laboratory of the Dana Farber Cancer Institute, Boston, MA).

### Luciferase reporter assay for p21expression

The p21 promoter-luciferase reporter plasmid was constructed using a 2.4 kB fragment from the upstream region containing OLIG2 binding sites in the human p21 promoter, and was a gift from Charles Stiles of the Dana Farber Cancer Institute. The construct was co-transfected with control vector pCDH-CMV-MCS-EF1-Green-Puro or one encoding the human OLIG2 ORF using Purefection (System Biosciences, Mountain View, CA) into 293FT cells. In some cell preparations 10 μM OLIG2 inhibitor compound was added. Cells were harvested and assayed for luciferase activity 24 h after transfection using a luciferase assay kit (Promega, Madison, WI) and an Infinite^®^ M1000 PRO plate reader (Tecan, Morrisville, NC) for luminescence quantification.

### Fluorescence-based thermal shift assay (TSA)

TSA was used to test affinity of the inhibitor SKOG102 to recombinant OLIG2 protein. The assay was conducted in the 96-well-based CFX-96 real-time fluorescence plate reader (BioRad, Hercules, CA). The fluorescent dye Sypro Orange (Sigma, St. Louis, MO) was used to monitor the protein folding-unfolding transition. Protein-ligand interaction was gauged by shift in the unfolding transition temperature (ΔTm) acquired with protein alone or with protein in the presence of the inhibitor. Each reaction sample consisted of 9.8 μM OLIG2 protein mixed with 0, 12.5, 25, or 50 μM inhibitor in 1% DMSO, and incorporated 3X Sypro Orange dye in 20 μl reaction buffer (25 mM Tris-HCl pH 7.5, 5 mM MgCl_2_, 0.5 mM DTT, 2.5% glycerol). The sample plate was heated from 25°C to 95°C with a thermal ramping rate of 1°C/min. The fluorescence signals were acquired with excitation and emission wavelengths centered at 490 and 560 nm, respectively. Affinity (KD) was calculated based on the degree of fluorescent shift in protein melting temperature (Tm) with and without the inhibitor [44].

### EMSA studies

GBM8 glioma patient-derived cells were treated with Olig2 inhibitor at 5 μM or DMSO vehicle control for 20 hrs. Nuclear and cytoplasmic fractions were then isolated using ActiveMotif Nuclear Extraction Kit (Cat No. 40010). Nuclear extracts were used for electrophoretic mobility shift assay (EMSA). To detect Olig2 binding DNA the Gelshift Chemiluminescent EMSA Kit from ActiveMotif was used (Cat No. 37341). The 27 bp oligonucleotide sequence (sense strand 5′-gctcagagcccagctgctggactgagc -3′) with Olig2 binding site (bold and underlined) was synthesized by IDT and contained a biotin tag at the 5′terminus of both strands. EMSA was performed according to kit instructions; nuclear extract (5 ug of protein) was incubated for 20 minutes at room temperature with 20 fmol biotinylated DNA. Nuclear and cytoplasmic extracts were subjected to immunoblot analysis with antibodies to Olig2 (Dana-Farber Ab#308), PhosphoSerine 10,13,14-Olig2 (Abcam), Lamin A/C (ActiveMotif), E47 (E2A) (Cell Signaling Technology), Tubulin (Sigma).

### *In vivo* studies ethics statement

All animals were handled in accordance with guidelines set forth by the National Institute of Health [45, 46] and the Principles for the Use and Care of Vertebrate Animals [47]. Further, all the animal work described herein was reviewed and approved by the University of California San Diego Institutional Animal Care and Use Committee (IACUC).

### *In vivo* GBM flank xenograft tumor model

2 × 10^6^ GBM4 cells were implanted subcutaneously in flanks of NSG mice. When tumors were measurable at 50-110mm^3^, treatment was initiated. SKOG102 was dissolved in solutol:PEG400:water (20%:40%:40%), injected intraperitoneally. Dosing schedule was as follows: The first 2 week period (from day 33 to 46) 10 mg/kg, the third week (from day 47 to 54) 15 mg/kg, fourth week (from 55 to 66) first 3 days 20 mg/kg, 2 days break, then alternate days from day 60 to 66. Control mice were treated with vehicle control on same schedule (*n* = 5). Tumors were measured using calipers and their volume was calculated using the standard formula, V = (length × width^2^)/2, *p* = 0.02.

### Brain entry of SKOG102 inhibitor

SKOG102 at 5 mg/kg was injected intraperitoneally in 6 healthy, intact balb/c mice. Then at 1 and 4 hours after injection the mice were deeply anesthetized and blood samples were removed via cardiac puncture, and the brains were dissected free. Brain sections were analyzed by MALDI mass spectrometry imaging (MSI) to visualize brain entry of SKOG102. LC-Mass spec was used to determine plasma and brain concentrations of SKOG102 expressed as ng/mL plasma and ng/mL (ng/gm) of brain tissue.

### *In vivo* intracranial tumor model

Primary human patient derived glioblastoma cancer stem cells (GBM4) were dissociated, plated, and treated immediately *in vitro* with either OLIG2 inhibitor (5 μM) or DMSO for 14 h. Cells were then manually dissociated, a sample withdrawn, and the proportion of viable cells was assessed by trypan blue exclusion. The OLIG2 inhibitor/DMSO treated cells were resuspended in HBSS at a concentration of 60,000 viable cells/μL, and 5 μL was injected into the right striatum of SCID mice under stereotaxic control (*n* = 8). Four weeks after tumor inoculation MRI imaging of the brain was performed with a 1.5-cm custom-built manually tuned surface MRI coil, and a horizontal-bore 7T MRI scanner (General Electric Co., Fairfield, CT).

### Statistical analysis

Values are expressed as the mean ±standard deviation. Individual comparisons were performed using the two-tailed Student's *t* test. *P* values < 0.05 were accepted as significant.

## SUPPLEMENTARY MATERIALS FIGURES


