# Supplementary material for: Multiple spatially related pharmacophores define small molecule inhibitors of OLIG2 in glioblastoma
Source: Oncotarget. 2015 Oct 30;8(14):22370–84. doi: 10.18632/oncotarget.5633 (PMC5410230; doi:10.18632/oncotarget.5633)
Supplement: Supplementary file 1 [file oncotarget-08-22370-s001.pdf]

# Multiple spatially related pharmacophores define small molecule inhibitors of OLIG2 in glioblastoma

**Supplementary Material**

**Methods**

**RT-PCR:** Cancer stem-like cells were isolated from patient GBM tissue samples and cultured in NSA proliferation medium (Stemcell Technologies, Vancouver, Canada). After 72 h incubation with control shRNA or OLIG2 shRNA, or after 12 h of incubation with DMSO control or OLIG2 inhibitor compounds, cells were harvested and mRNA was extracted with the AllPrep DNA/RNA Mini Kit (Qiagen, Inc., Valencia, Calif.), followed by cDNA synthesis using the iScript cDNA Synthesis Kit (Bio-Rad, Inc., Hercules, Calif.). To investigate single-gene expression patterns, individual gene primers were selected from PrimerBank (http://pga.mgh.harvard.edu/primerbank/) and then were purchased from Allele Biotechnology and Pharmaceuticals Inc. (San Diego, Calif.). SYBR Green Real Time PCR master mixes were purchased from Roche Corporation (Roche New Jersey, Nutley, N.J). RT-PCR was performed with primers specific for human OLIG2 and P21 specific primers.

Immunoblotting: GBM cells were transfected with Scr ShRNA aor OLIG2 ShRNA. Cells were collected and washed with PBS, lysed with RIPA buffer (Pierce & Co., Nashville, Tenn.) containing protease inhibitor cocktail. Proteins were quantitated by the BCA protein assay (Bio-rad). Equal amounts of protein were resolved by polyacrylamide gels, transferred to nitrocellulose membrane and probed with OLIG2 antibody obtained from Charles Stiles's laboratory of the Dana Farber Cancer Institute (Boston, Mass.).

**Mass spectrometry imaging analyses for entry of SKOG102 into the mouse brain:** The mice were injected intraperitoneally with 20 mg/kg SKOG102 and the brains dissected free and stored at -25°C 4

hours after injection. Tissue sections were prepared using a Microm HM550 cryostat (Thermo Scientific™, Waltham, MA, USA) with the microtome chamber chilled at -20°C and the specimen holder at -19°C. The tissue was sectioned at 12 μm thickness in the coronal plane, and thaw mounted onto ITO-coated glass slides (Bruker Daltonics, Billerica, USA) for MALDI MSI, and onto optical slides for hematoxylin and eosin (H&E) staining. Samples were dried for 15 minutes in a desiccator. α-Cyano-4-hydroxycinnamic acid (CHCA, Sigma-Aldrich, Saint Louis, MO, USA) was deposited using the ImagePrep (Bruker Daltonics, Billerica, USA) at a concentration of 5 mg/mL in ACN/0.2% TFA 60:40 vol/vol. Mass spectra were acquired with the UltrafleXtreme MALDI-TOF/TOF mass spectrometer (Bruker Daltonics, Billerica, USA) equipped with a 1 kHz smartbeam laser. MALDI MSI experiments were acquired with a pixel step size set to 75 µm in FlexImaging 4.0 software (Bruker Daltonics, Billerica, USA). Spectra were externally calibrated using a custom calibration standard solution for small molecule calibration. Spectra were acquired in the positive ion mode from 1000 laser shots accumulated at each spot for a mass range *m/z* 0-1000. The laser intensity was set to 30% with a frequency of 1000 Hz. The MALDI images were displayed using FlexImaging 4.0. The permeability of CT-102 through the blood vessel is visualized following the signal of the drug (*m/z* 422.2 ± 0.2) and heme (*m/z* 616.2 ± 0.2) as previously described.

MSMS analyses of CT-102 from a 1:1 mixture of CT-102 standard solution (10 μM in $H_2O$) with CHCA (5 mg/mL in ACN/0.2% TFA 60:40 vol/vol), and from prepared brain tissue sections were performed using the LIFT™ mode. The acceleration voltage was 8 kV in the MALDI ion source and 19 kV for fragment postacceleration in the LIFT cell. The precursor ion (*m/z* 422.2) selector resolution was set to 1%. A total of 5,000 shots were accumulated for each mass spectrum at a laser intensity set to 50%.

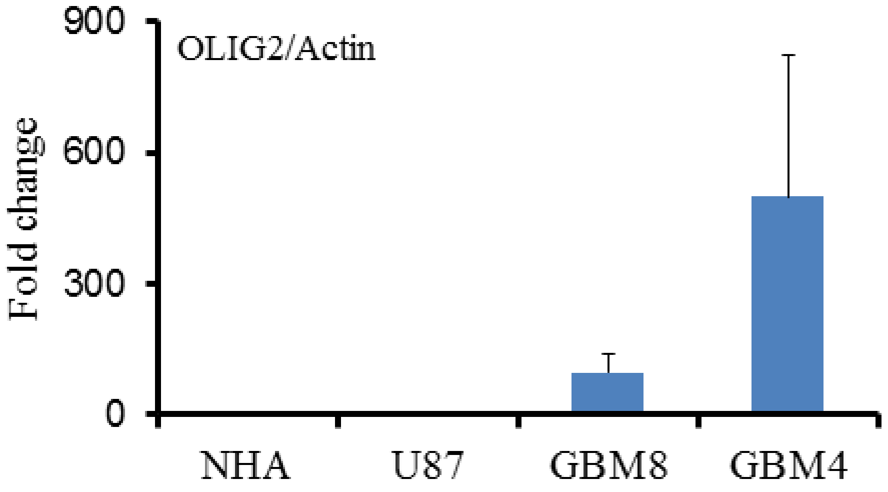


**Fig. S1. OLIG2 expression for GBM cell lines and normal human astrocytes.**

RT-PCR identifies OLIG2 expression relative to actin for patient-derived GBM lines (GBM4 and GBM8), for a serum-grown GBM cell line (U87) and for normal human astrocytes (NHA) freshly acquired from patient material. Although not indicated by the scale, more OLIG2 is expressed by U87s than by NHAs.

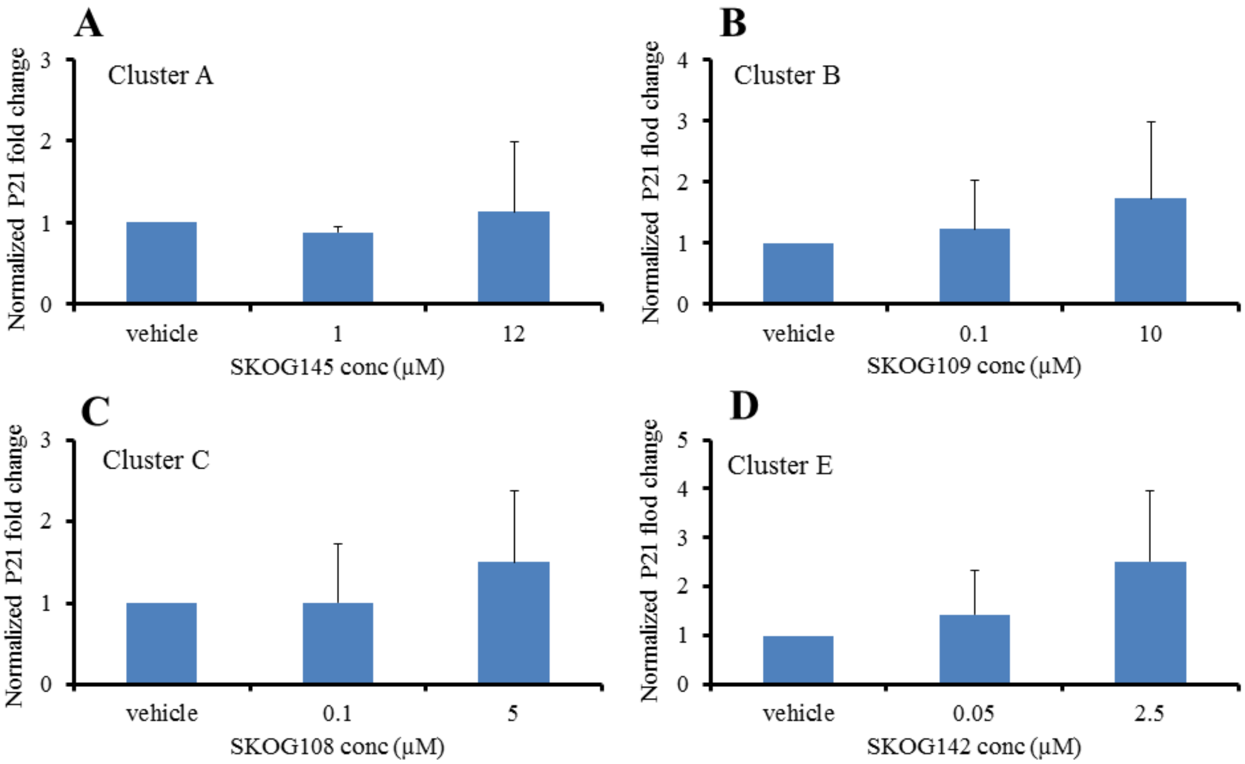


**Fig. S2.** **P21 expression with putative OLIG2 inhibitors**

To determine whether the OLIG2 inhibitors from different clusters affect downstream targets of OLIG2, GBM4 cells were incubated for18 h with inhibitor compounds (SKOG145, SKOG109, SKOG142 and SKOG108) at two different doses, and expression of p21 was determined by RT-PCR. Escalating OLIG2 inhibitor doses led to increased levels of p21. (***A***) SKOG145 (Cluster A). (***B***) SKOG109 (Cluster B). (***C***) SKOG108 (Cluster C). (***D***) SKOG142 (Cluster E). P values are not significant for this experiment at the doses used.

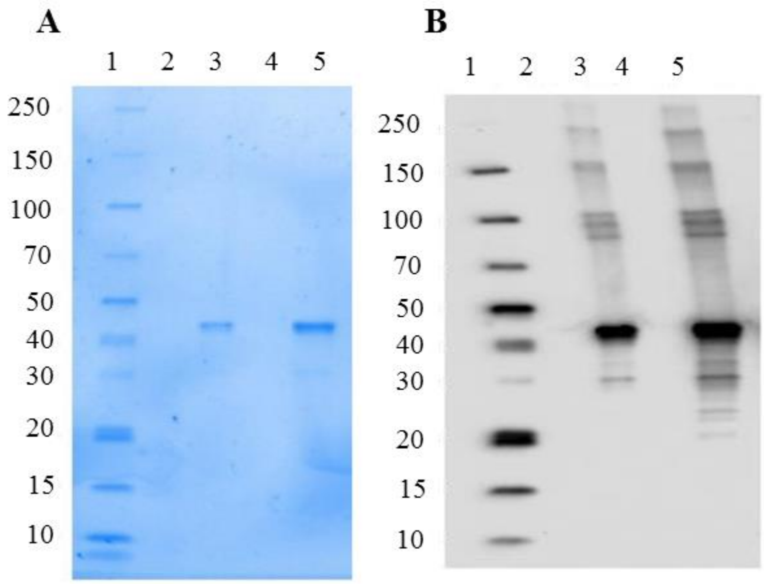


**Fig. S3. Analysis of His tag -OLIG2 by reducing SDS-PAGE.**

Protein obtained from Blue Sky. Different volumes of 0.53 mg/ml concentration of purified Protein from E.coli were loaded in to SDS-PAGE (lane 3 and lane 5). A) Coomassie blue staining: B) staining with anti-polyHistidine antibody.

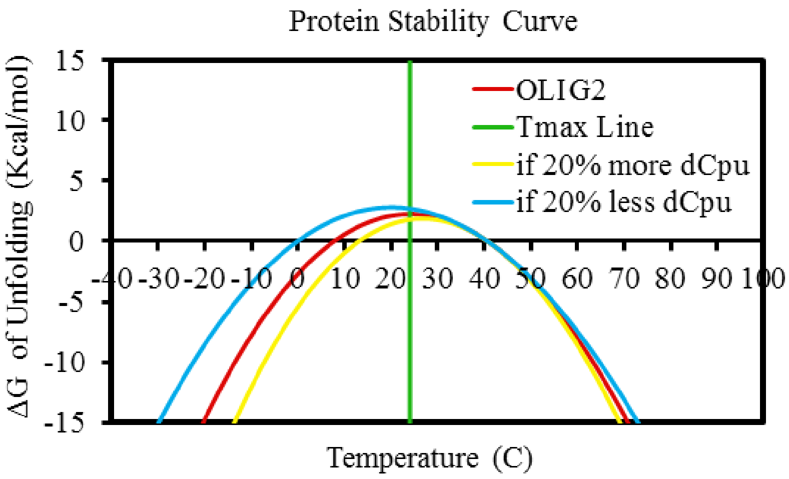


**Fig. S4. Estimated free energy (ΔG) of unfolding of OLIG2 protein.**

Tmax is the predicted maximal thermal stability of OLIG2 under used buffer conditions. The stability curves are estimates only based on the melting profile of the protein, with an arbitrary 20% uncertainty in calculated $\Delta C_{pu}$ (heat capacity change of unfolding).

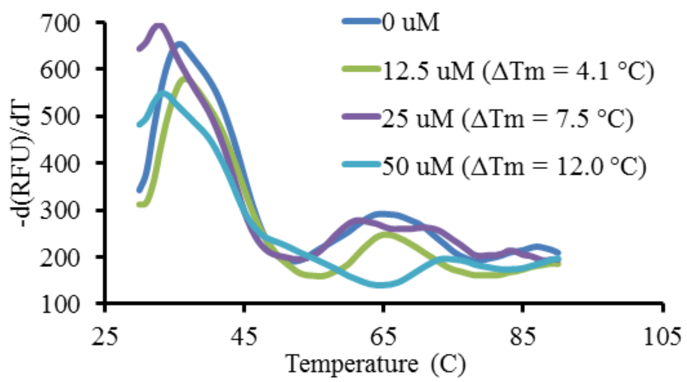


13

**Fig S5. Biophysical measure of SKOG102 binding to OLIG2.**

Melting profile of OLIG2 protein as measured by fluorescence-based thermal shift assay with and without SKOG102. OLIG2 protein melts with an average Tm of 52.0°C in the inhibitor-free state, and with Tm of 52.0°C, 59.5°C and 64°C when 12,5 µM, 25 µM and 50 µM inhibitor is added, respectively. The results indicate addition of higher concentration of the inhibitor stabilizes the protein as reflected with increasing Tm. ΔTm is measured as 4.1°C, 7.5°C and 12.0°C at 12,5 µM, 25 µM and 50 µM of added inhibitor, respectively.

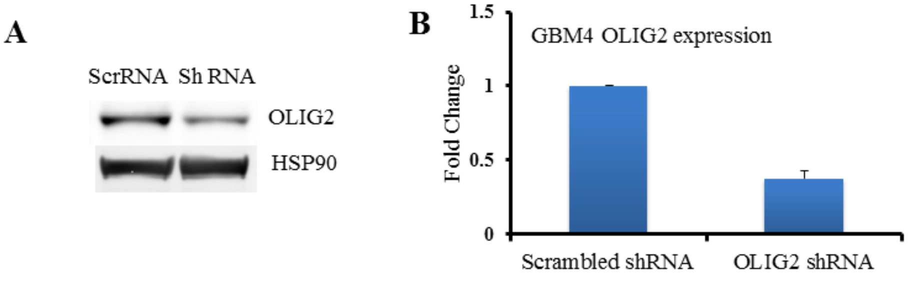


**Fig. S6. Knockdown of OLIG2 in GBM4 cells.**

GBM4 cells were transfected with OLIG2 shRNA and scrambled shRNA, cells were harvested, run western blot and RT-PCR to check levels of OLIG2. Fold change is with respect to actin.

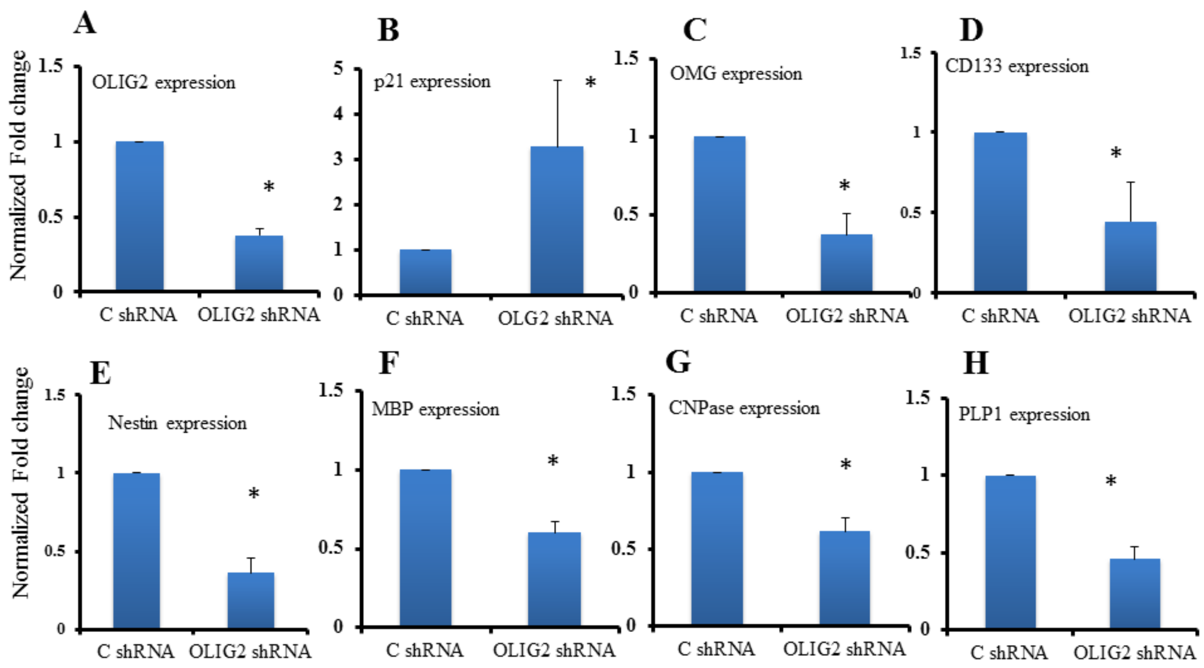


**Fig. S7. Effect of OLIG2 knockdown on oligodendrocyte cell lineage markers.**

OLIG2 knockdown decreased expression of surface markers of oligodendrocyte lineage and stem cells. RT-PCR analyses of GBM stem-like cells (GBM4) derived from human primary tumors treated with OLIG2 shRNA to indicate the expression of oligodendrocyte markers (OMG, CNPase, MBP, and PLP1) and stem cell markers (CD133 and Nestin) on GBM stem-like cells n=5 per experiment).

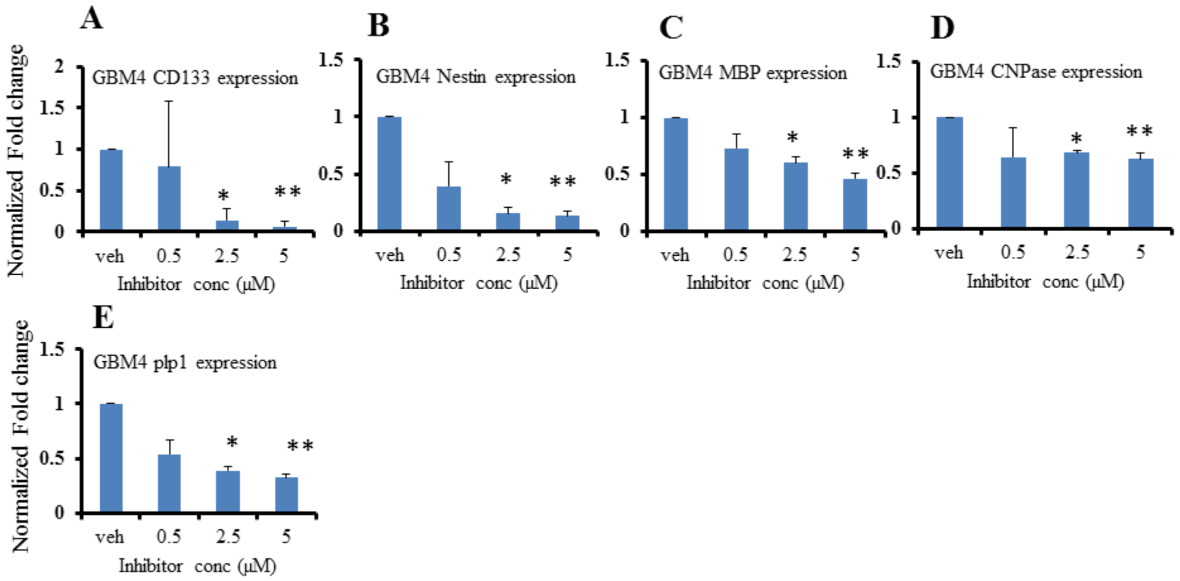


**Fig. S8. Effect of SKOG102 on expression of oligodendrocyte lineage markers.**

OLIG2 inhibitor compound decreased expression of surface markers of oligodendrocyte lineage and stem cells. GBM4 cells treated with the OLIG2 inhibitor compound duplicated the results on lineage marker expression obtained with OLIG2 shRNA, and showed dose dependency. Data shown is the mean of duplicates for each test. Error bars represents mean standard deviation, * indicates *p*-value between vehicle and 2.5 µM inhibitor treated cells ($p < 0.05$) and ** indicates *p*-value between vehicle and 5 µM inhibitor treated cells ($p < 0.01$).

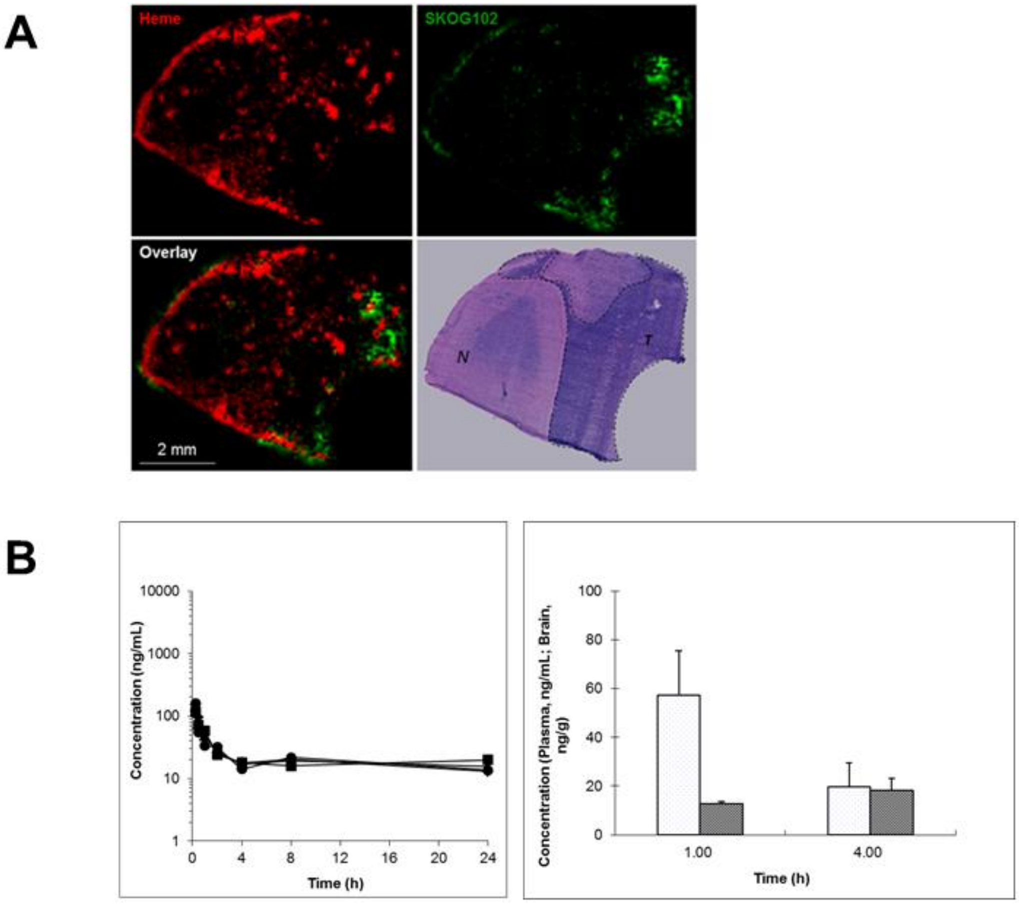


**Fig. S9 SKOG2102 enters orthotopic GBM tumors and the brain**

**A. SKOG102 entry into orthotopic GBMs.**

To evaluate SKOG102 delivery, mice with a tumor xenograft were treated with a single dose of SKOG102 and brain sections were analyzed by MALDI mass spectrometry imaging (MSI). Imaging data indicated that SKOG102 is accumulating in the tumor region. MALDI-TOF MS images of a mouse brain section with an orthotopic tumor with 4-hours single intraperitoneal dose of 20 mg/kg SKOG102. Red and green colors indicate the distributions of heme (*m/z* 616.2 ± 0.2) and CT-102 (*m/z* 422.2 ± 0.2), respectively. Yellow color indicates overlay of heme and SKOG102. Black dotted lines delineate tumor tissue (‘*T*’) from normal tissue (‘*N*’) in the H&E stained sister section.

**B. OLIG2 small molecule inhibitor (SKOG102) has favorable PK and crosses BBB making it feasible for *in vivo* tests. Left Panel:** the plasma concentration of inhibitor after 5 mg/kg IP injection in balb/c mice, 3 trials. By 4 hours concentration is at steady state which is long-lived. **Right Panel:** The brain concentration of SKOG102 at 1 hour and at 4 hours after 5 mg/kg IP injection. Note at 4 hours plasma concentration is matched by brain concentration. **Open bars** = plasma (ng/mL); **Closed bars** = brain (ng/g). N=3 per time point.

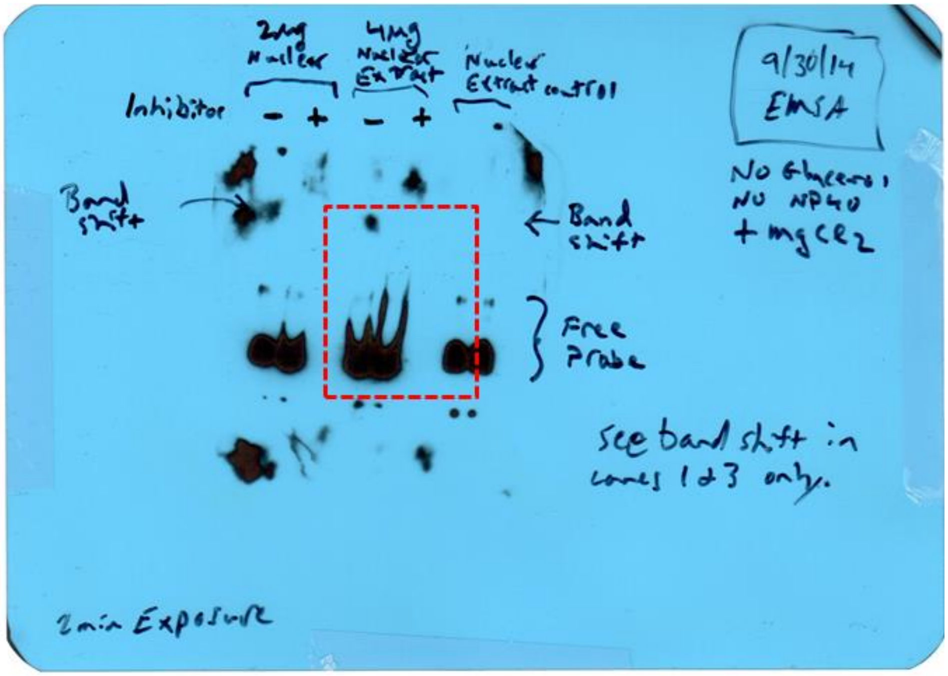


**Fig. S10. Full image of Western blot shown in Figure 7A.**

Red box indicates cropping lines.

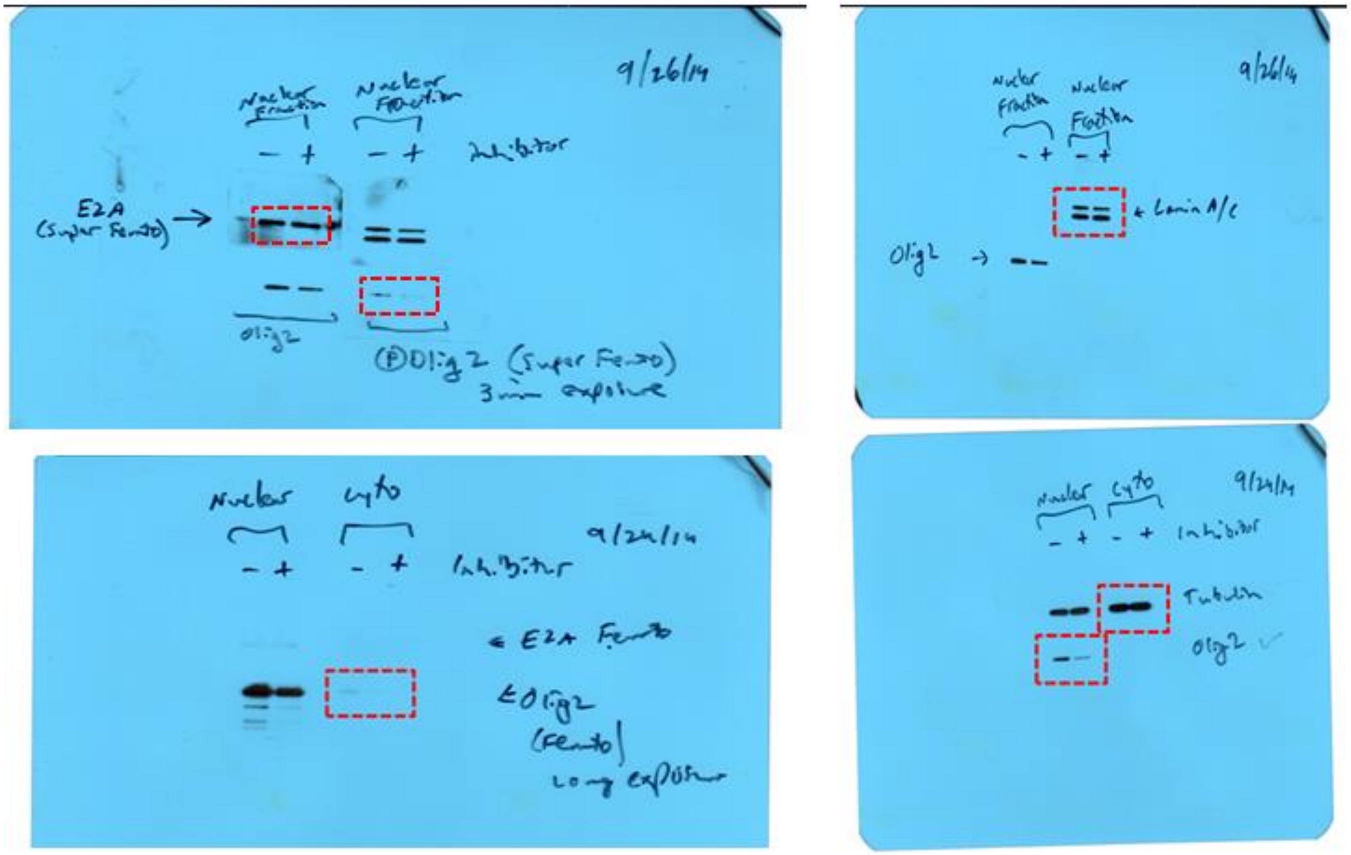


**Figure S11. Full images of blot panels shown in Figure 7B.**

Red boxes indicate cropping lines.
